# Farmland Transfer, Social Security, and Households’ Productive Investment: Based on China’s CFPS Survey

**DOI:** 10.3390/ijerph191711082

**Published:** 2022-09-04

**Authors:** Shangan Ke, Yueqi Wu, Haiying Cui, Xinhai Lu, Danling Chen

**Affiliations:** 1 School of Public Administration, Central China Normal University, Wuhan 430079, China; 2 Institute of Natural Resource Governance, Central China Normal University, Wuhan 430079, China; 3 College of Public Administration, Huazhong University of Science and Technology, Wuhan 430074, China; 4 College of Public Administration, Huazhong Agricultural University, Wuhan 430070, China

**Keywords:** farmland transfer, social security productive investment expansion, CFPS

## Abstract

The willingness of farmers to transfer land on a big scale will be impacted when the rural social security system is not ideal, which will limit households’ productive investment. This paper investigated the intermediate effects of social security on farmland transfer and productive investment by using zero-inflated models based on 4703 samples across China. Here are the findings: (1) Farmland transfer does not significantly impact productive investment without considering social society. (2) With the improvement in social security, farmland transfer significantly affects fixed investment but is not the same as households’ current investment. (3) Under the social security constraints, there is an inverted U-shaped relationship between farmland transfer and current investment. (4) The partial effect of farmland transfer on fixed investment is significantly positive, and it shows a trend of rising volatility. The government should re-examine the expected effects of the farmland transfer policy and focus on the farmers’ worries about the future. Meanwhile, it is necessary to comprehensively improve the social security system and improve the multi-dimensional survival ability of farmers to give full play to the critical role of farmland transfer in current investment.

## 1. Introduction

Sound agricultural productive investment can recombine, adjust production, and increase labor productivity. It is one of the leading factors driving agricultural growth. However, the slowdown in China’s agricultural development mainly results from exhausting the benefits of the reforms’ incentive effect, restricting farmers’ willingness to invest in production. Incentivizing the productive investment of Chinese farmers is vital to modern agricultural development. Farmland transfer is a choice to promote productive investment and develop modern agriculture. It is defined as the transfer of contractual management rights over farmland through leasing, exchange, or subcontracting following the law and is characterized by a change in the scope, means, and uses of farmland in The Rural-Land Contract Law of the People’s Republic of China. Farmland transfer can realize the aggregation of production materials, expand production scale, increase production efficiency, and realize economies of scale by transferring the right to other farmers or economic organizations.

However, China’s farmland transfer faces significant challenges. According to data released by the Ministry of Agriculture and Rural Affairs of China (http://zdscxx.moa.gov.cn, accessed on 1 July 2021), in 2016, 2017, and 2018, the transferred area of rural contracted land in China was 32 million, 34 million, and 35 million hectares, respectively. From 2014 to 2017, the area of land transferred increased by 18.3%, 10.8%, 7.2%, and 6.9%. The farmland transfer has shown an “involute” trend, although the whole scale is uprising. More than 63% of China’s farmland is still in the hands of farmers and has not been transferred. The slowdown in the growth of farmland transfer is becoming more and more apparent. The weakening of the willingness to transfer, the limited transfer scale, and the low level of investment are the three core issues currently facing China’s farmland inflow. The transfer of China’s farmland may fall into the bottleneck of the “The Moral Economy of the Peasant.” American anthropologist Scott first proposed the Moral Economy of the Peasant theory, which essentially reflects the survival rationality of farmers under the product economy with self-sufficiency as the main feature. The theory believes that small farmers’ adherence to “safety first” and “livelihood first” can explain farmers’ production activities, social arrangements such as exchange and reciprocity, and behavioral choices such as resistance.

Based on this theory, we believe that China’s farmland transfer obstacles lie in the lack of farmers’ sense of security, which is essentially the risk aversion behavior of small farmers under the mode of Moray Economic behavior. The research shows that the vague rural land property rights, the unsound market for farmland transfer, and farmers’ land complex are the leading causes affecting land transfer, especially in the current rural areas in China. Significantly, the unsound social security system has become a restrictive factor restricting farmland transfer (Gao et al., 2017; Hong and Luo, 2018; Gao et al., 2013) [1,2,3]. The factors mentioned above can be regarded as farmers’ worries about the uncertain risks of future survival and development and a manifestation of the lack of “social security” in a broad sense. 

In addition, existing research has focused on the impact of farmland transfer on expanding productive investment and believes the value of farmland transfer is to increase households’ income to strengthen productive investment by increasing the handled farmland area. However, suppose farmers are unwilling to engage in farmland transfer because of risks such as low agricultural income or inadequate social security and reluctance to invest after farmland transfer. In that case, farmland transfer will not achieve productive investment expansion. There is not plenty of evidence to support farmland transfer to promote productive investment. For example, according to China Family Panel Studies (CFPS) data in 2018, the average investment for the transferred farmland is nearly CNY 1500, lower than the CNY 1800 of the national high-standard farmland. Under an imperfect rural social security system, farmers do not have enough reasons to voluntarily transfer land contractual management rights on a large scale to avoid expanding productive investment and irrational loss. It is not rigorous enough to assume that farmland transfer can directly extend productive investment.

From this belief, this research focus on the connections between farmland transfer and productive investment expansion based on the Moral Economy of the Peasant theory. In addition, the paper explored the intermediate effects of social security on their connections based on 4703 samples covering 24 provinces, 121 counties, and 310 villages across China with China Family Tracking Studies (CFPS) of 2010, 2014, and 2018. The main contribution of this paper is to explore how farmland transfer and social security influence the behavioral choices of households’ productive investment and verify whether the behavioral decisions of farmers in farmland transfer conform to The Moral Economic of the Peasant theory. The research also provided new evidence for China’s farmland transfer theory and policy implications. Additionally, it offers a theoretical foundation for encouraging farmland transfer, enhancing the social security system, and motivating households’ productive investment.

## 2. Literature and Hypothesis

Farmland is fundamental to most farmers’ living. Using land properly is of great significance to promote agricultural development. In addition, the influence of farmland transfer on the productive investment of farmers is the core driving force of agricultural development. In recent years, the impact of farmland transfer on the productive investment of farmers has been widely concerned by scholars, but the results are controversial. The imperfect farmland transfer market on the productive investment of farmers is not necessarily positive [4]. Gao et al. [5] found the probability and amount of organic fertilizer on the transferred land are less than their own. It indicates that hastening farmland transfer may also harm the productive investment of farmers. Xu et al. [6] found that small-scale farmland transfer is not enough to increase farmers’ total income significantly, nor enough to make it have the incentive to increase the productive investment of farmers. However, most scholars hold a relative opinion. They believe that farmland transfer expands land scale, which is conducive to increasing households’ productive investment [7,8].

There are two critical influence mechanisms: first, the farmland transfer influences the productive investment of farmers by increasing the chance of households’ credit. Farmland transfer can significantly improve farmers’ loans and agricultural investment. Promoting farmland transfer can encourage farmers to use loans for agricultural production. The scale of farmland will dramatically impact farmers’ borrowing, and farmers with a large land scale can receive more loans [9]. Second, farmland transfer affects the productive investment of farmers by increasing the capital accumulation, which is mainly manifested in increasing farmers’ income. For a long time, the productive investment of the farmers depends on primitive accumulation and household income to a great extent. Farmland transfer is conducive to increasing farmers’ income, which is conducive to improving rural residents’ ability of making capital investment [10].

The productive investment of farmers can be divided into current and fixed investments, and some scholars believe farmland transfer impacts both types of investment. However, some research finds that the impact of farmland transfer on the two types of investments may differ [11]. It can promote the current investment but does not regulate the fixed investment [12]. To verify the above conclusions, we think both investment types have an expansionary effect on farmers’ fixed asset investment. Based on the above analysis, the following assumptions are made:

**Hypothesis** **1.** *Farmland transfer can effectively promote the growth of the current productive investment of farmers*.

**Hypothesis** **2.** *Farmland transfer can effectively promote farmers’ productive fixed investment growth*.

As China’s economy grows, millions of farmers are hungry for sound social security. Because of low agricultural income and an imperfect social security system, farmers are reluctant to transfer farmland. It may affect the productive investment of farmers. For example, in the study of agricultural mechanization development in the United States, it was found that low subsidy levels were not attractive to farm household investors [13]. The indirect impact of farmland transfer on productive investment through enhancing social security is as follows. As a bridge, the relationship between farmland transfer and social security has aroused the close attention of some scholars. According to academics, social security encourages farmland transfer and raises transfer rent, encouraging farmers to increase farmland investments. [14,15]. Through field research, Zheng et al. [16], with actual data, confirmed that the lack of social security will slow down the speed and scale of farmland transfer. However, there is a close relationship between farmers’ investment willingness and social security. Some scholars have found it is of great significance to perfect the farmland transfer market and regulate the behavior of farmland transfer for guiding different types of farmers to make the sensible investment in agricultural land, promoting the sustainable use of farmland, increasing farmers’ income and ensuring national food security [17]. Based on the above analysis, the following assumptions are made:

**Hypothesis** **3.** *Through the regulation of Social Security, farmland transfer has a significant impact on the current productive investment of farmers*.

**Hypothesis** **4.** *Through the regulation of Social Security, farmland transfer has a significant impact on the productive fixed investment of farmers*.

The most important is how to achieve a positive interaction of farmland transfer, social security, and productive investment of framers. To sum up, scholars have conducted much research on farmland transfer. Existing studies have focused on the relationship between farmland transfer and productive investment of farmers or between farmland transfer and social security. However, the studies that the level of rural social security may regulate farmland transfer and productive investment of farmers have not been performed. This paper aims to study the regulation of social security in farmland transfer and productive investment of farmers, enrich the existing research on farmland transfer, and provide theoretical support for future examples of farmland transfer.

## 3. Methodology and Data

### 3.1. Model Setting and Zero-Inflated Treatment

To explore the effects of the land transfer on the productive investment of farmers, we first set a basic regression equation, and the expression is as follows:

(1) $${\mathrm{invest}}_{i}=\alpha_{0}+\alpha_{1}{\mathrm{socialsc}}_{i}+\underset{n=1}{\sum }\alpha_{2}X_{ni}+\varepsilon_{i}$$

In Equation (1), *inwest_i_* represents the household investment of farmers, including current investment and fixed-asset investment. *socialsc_i_* is the indicator of social security, weighted calculation with four dimensions of non-agricultural economy, social network guarantee, social system guarantee, and commercial insurance. *X_ni_* is the control variable set, including family endowment, farmland endowment, and community attributes. *α*_0_ is the constant while *α*_1_ and *α*_2_ are the coefficients for core independent variable and control variables. *ε_i_* is the error term. We use the robust Ordinary Least Square (OLS) to estimate Equation (1).

Based on Equation (1), this paper further explores the impact of social security on farmers’ productive investment behaviors under the realistic background of farmland transfer. Therefore, the variable of social security is introduced in Equation (1) to analyze whether social security can affect farmland transfer, thereby affecting farmers’ productive investment expansion. In addition, since the farmers’ willingness to invest in productive investment at the current stage is not strong, the dependent variables of current and fixed investment have over 60% zero outcomes. To solve the zero-inflated problem, we use the zero-inflated Poisson regression model to fit the result. Here is the equation:

(2) $$P\left(y_{i}|x_{i},z_{i}\right)=\{\begin{array}{c} \theta_{i}+\left(1-\theta \right)exp\left(-\lambda_{i}\right),y_{i}=0\\ \left(1-\theta_{i}\right)\frac{\lambda_{i}^{y}}{y!}exp\left(-\lambda_{i}\right),y_{i}>0 \end{array}$$

In Equation (2), *y_i_, x_i_, z_i_* are the independent variable, dependent variable, and the inflated variable, respectively. The parameter *θ* is the probability of the dependent variable *y* reaching zero, while 1 − *θ* is the probability of *y* reaching nonzero positive integers. Let $\lambda =\exp\left(x_{i}^{\prime }\beta \right)$ and substituting to Equation (2), and we will obtain the zero-inflated Poisson regression model. The parameter *θ* (usually called the zero-inflated parameter) represents the proportion of non-Poisson data with a value of 0 (also called structural 0). In this model, the choice of independent and dependent variables is the same as Equation (1). In the hypotheses, (3) and (4) of this paper, the social security level is the cause of many zeros in productive investment variables, so the social security level is set as an inflated variable.

### 3.2. Variables Selection

Productive investment. Farmer’s productive investment can be divided into current investment and fixed investment. Current investment refers to investment directly related to farmland, including seeds, pesticides, fertilizers, while fixed investment refers to the investment not directly connected to farmland, mainly referring to the input of agricultural machinery. Current investment is represented by the monetary value of seeds, fertilizers, pesticides, hired workers, machine leasing, and irrigation that farmers put into agricultural production in 2018. Fixed investment is represented by the total value of various agricultural machinery owned by farmers in 2018. Considering the significant differences in farmland owned by farmers in different regions, the average current investment (fixed investment) is calculated by dividing the current investment (fixed investment) by farmland area operated by the farmer, and logarithmic processing is performed separately.

Farmland transfer. The farmland transfer is divided into two types: farmland inflow and farmland outflow. Generally speaking, the transfer of agricultural land will lead to the expansion of farmers’ productive investment, and the transfer of agricultural land will cause the contraction of productive investment. This article focuses on studying the inherent relationship between the transfer of agricultural land and farmers’ expansion of productive investment. Therefore, the “total scale of household inflows into farmland” is selected to characterize the transfer of agricultural land, including paddy fields, pastures, ponds, paddy fields, orchards, types of agricultural land such as forest land, and z-standardized variables.

Comprehensive social security. Previous studies have mainly used a single dimension such as social security and commercial insurance to measure farmers’ social security levels. To measure the level of social security of farmers more comprehensively, we divide social security into non-agricultural economic security, social relationship security, social system, and commercial insurance. The non-agricultural economic security is mainly used to measure the capability of acquiring income in addition to agricultural production, including the population proportion of out-working and the income proportion from out-working. Social relations security measures the strength of the family’s social network, characterized by the social-contact expenditure. Social system security measures the public services of social security, represented by participation in rural cooperative medical care and government transfer payments. Commercial insurance includes commercial and family medical care expenditures in the past year. After preprocessing the above indicators, the entropy method calculates farmers’ comprehensive social security level. Proportion of out-working, income from out-working, whether the salary income exists, the salary sent home, social-contact expenditure, years of participation in rural cooperative medical, government transfer payments, and family medical care expenditures in the past year are calculated according to the entropy method. After that, the weight ratios are 0.0767, 0.0923, 0.1118, 0.0598, 0.0103, 0.2618, 0.3088, and 0.0785.

Control variable set. In addition to the above three key variables, this paper also controls 17 variables such as the endowment of farm households, the endowment of farmland, and the village environment. At the same time, the variable “scale of farmland outflow” is also selected as the control variable. The reason is that, on the one hand, after the land transfer, the scale of farmer households’ operation becomes smaller, their unit production costs increase, and farmers’ enthusiasm for participating in agricultural production may decrease. The behavior is not conducive to productive investment; on the other hand, after the transfer of farmland, labor is transferred to non-agricultural industries, household income increases, and investment capacity improves. Family endowments include the degree of a family’s emphasis on education, agricultural income structure, and population size. Farmland endowments include the family’s per capita arable land scale, total land value, and land structure. The village environment includes the village’s economic development, location, and industrial structure (Table 1).

### 3.3. Data Source and Pretreatment

This paper is based on the China Family Tracking Studies (CFPS) of 2010, 2014, and 2018, mainly including three dimensions: individuals, families, and communities. It investigates detailed data about rural households’ farmland transfer, productive investment, and social security. The CFPS sample covers 25 provinces (autonomous regions, municipalities) across China and adopts a three-stage overall sampling method with unequal probability. The population of the investigation area accounts for about 95% of China’s total population, which is nationally representative. To fully reflect the relationship between social security, farmland transfer, and farmers’ productive investment during the transition period, the empirical part uses the recently updated CFPS2018 data.

This paper first screened a sample of rural households with registered permanent residence in the data processing. Then, according to the research objectives and CFPS item settings, we selected the primary variables, including variables that characterize social security, farmland transfer, farmers’ productive investment, and control variables, which specifically cover family endowment characteristic variables, land endowments, and villages’ economic and social characteristics. Due to the lack of village data in CFPS2018, we integrated family data in CFPS2010 and CFPS2018, merged with CFPS2014 community data, and selected samples of farmers who participated in the three studies. Finally, this paper treats samples containing “Do not know,” “Not applicable”, and singular values as missing values and removes them. After the above processing, 4703 valid samples are obtained in this paper, covering 24 provinces (regions and cities), 121 counties, and 310 villages across China. Compared with previous studies, the data in this paper are relatively new, with a large sample size, possessing the time characteristics of the transition period and representing the country’s overall regional characteristics.

## 4. Results

### 4.1. The Effect of Farmland Transfer on Farmers’ Productive Investment

Table 1 reports the disruption of variables.

Table 2 reports the OLS estimation results of farmland transfer on the productive investment of farmers. The second, third, and fourth columns report the impact of farmland transfer on the total productive investment per land, the current investment per land, and the fixed investment per land, respectively. The regression results in Table 2 show that farmland transfer has not effectively promoted current and fixed investment growth. This research result overturns research hypotheses 1, 2, and 3 for this paper, showing that farmland inflow does not necessarily cause an increase in the productive investment of farmers in China.

From the perspective of family endowments, the research results indicate that the older the oldest family member, the more significant the negative impact on the productive investment. The impact coefficients of this indicator on current investment per land and fixed investment per land are −0.1871 and −0.2477, respectively, significant at the 99% confidence level. On the one hand, this situation is related to the current generation of farmers in China’s rural areas who tend to work, and the agricultural production and decision makers are mostly the elderly. Furthermore, on the other hand, it may be inseparable from the deep-rooted awareness of risk aversion among the elderly for avoiding higher investment risk. The average fixed investment coefficient (−0.2477) is also proving to be greater than the current investment coefficient (−0.1871) because fixed investment generally has a more significant investment quota and higher risks than currently. The family size has a significant impact on the productive investment of farmers at the 99% confidence level, and the fixed investment coefficient (0.5063) is much higher than the current investment coefficient (0.3168), reflecting that the more abundant the labor force, the more willing the families to inflow farmland and increase productive investment. Land asset value is also significant at the 99% confidence level from farmland endowments. This variable is a hidden location variable. Generally speaking, the more developed the regional economy and the better the location of the farmland, the higher its value. Average land assets’ significant, profound connotation is that farmers are willing to further make productive investments to increase their profit in more economically developed areas. From the perspective of the village environment, the higher the proportion of migrant workers in the village is significantly negatively correlated with the productive investment of farmers, indicating that the outflow of labor has led to a lack of labor in agricultural production and significantly inhibited the farmland transfer in China. There are differences in significance for household relative income, education expenditure, and other variables on current investment and fixed investment, mainly related to the two kinds of investment. The research also shows that household expenditure structure, average farmland, and land structure do not significantly influence farmers’ productive investment decisions.

We have proven that farmland transfer does not necessarily lead to expanding the average productive investment, which is a dangerous signal for China. On the one hand, more than 76% of farmers prefer not to participate in farmland transfer to carry out self-sufficient, small-scale production, indicating that China’s farmland transfer may not achieve the goal of expanding productive investment in agriculture and developing modern agriculture. Nevertheless, is this true? Considering that the estimation error caused by the zero-inflated model may be neglected in the OLS estimation, we use the zero-inflated Poisson Regression to explore the role of social security in farmland transfer and the expansion of productive investment.

### 4.2. The Regulatory Effect of Social Security

To solve the zero-inflated problem in the OLS model, we use the zero-inflated Poisson regression of Equation (2) to fit the coefficients between farmland transfer and farmers’ productive investment. Based on the above analysis, we assume that the level of social security is the reason for the low willingness of farmers for farmland transfer and productive investment, so the comprehensive social security level (*comp_socialsec*) is the inflated variable.

On the whole, from the zero-inflated Poisson regression model with *ave_curtinvest* and *ave_fxdinvest* as the dependent variables and *inflow_farmland* as the primary explanatory variable, *inflow_farmland* has a significant impact on the productive investment of farmers, and the main control variables are essential. The Vuong statics are 125.47 and 113.12, far more significant than 1.96. Therefore, the standard Poisson regression is rejected, and the zero-inflated Poisson regression should be used (Table 3).

Table 3 shows that farmland inflow significantly impacts productive investment after introducing the social security level. The coefficient of current investment (*ave_curtinvest*) is 0.0056, not considerable, while the coefficient of fixed investment is 0.0366 at the 90% confidence level. The results show that comprehensive social security is a reasonable expansion variable that significantly impacts rural households’ current and fixed investments, whose coefficients are 0.0951 and 0.0707, respectively, significant at the 90% and 95% confidence levels. In addition, the cross variable of farmland inflow and comprehensive social security has significant relations with current and fixed investment at a 99% confidence level. The result fully shows that the level of social security is an essential intermediary variable that affects farmland transfer and thus restricts the productive investment of farmers. Therefore, how to improve the level of comprehensive social security in rural China is an essential factor in promoting farmland transfer and expanding productive investment. Hypothesis 3 has not been proven, and Hypothesis 4 has been verified.

In the context of social security, the impact of farmland inflow on current investment did not have a significant effect. The possible reason is that the inputs of chemical fertilizers, pesticides, and other productive investments as traditional agricultural production materials are closely related to maintaining farmers’ livelihoods. Regardless of the farmers’ social security level, whether the farmers participate in farmland transfer, farmers need to invest a certain amount of production materials such as fertilizers, pesticides, and seeds to ensure their basic survival needs until they are wholly separated from agricultural production. Therefore, the impact of farmland inflow does not affect productive investment significantly. Correspondingly, farmland inflow has a significant effect on fixed investment. One possible reason is that labor division in agriculture is within the peasant households and the entire society under the open factor (service) market. With the promotion of modern agricultural technology, the separability of agronomic links and the traceability of agricultural activities has increased the roundaboutness of agricultural production, and agrarian division has been expanded and deepened. The application conditions for agricultural machinery are relatively harsh, and the investment threshold is the pretty high access to farmers’ wealth and social security. Therefore, the higher the level of comprehensive social security for farmers, the more they tend to flow into more farmland and expand fixed investment.

On control variables, factors such as the relative income (relative_income), family size (*family size*), the scale of farmland operated by the family (*optd_farmland*), the road conditions in the village (*innder_road*), and the proportion of migrant workers in the village (*mig_work*) all significantly impact the current investment and fixed investment of rural households. The structure of household expenditure (*stru_expd*) and scale of farmland outflow (*outflow_farmland*) are entirely uncorrelated with productive investment, and other variables are significantly correlated with current investment or fixed investment of farmers.

### 4.3. The Partial Effect of Social Security

It should be pointed out that although the above analysis can identify the adjustment effect of social security on farmland transfer, this adjustment effect is based on the conclusion obtained by the regression of the mean and cannot determine the adjustment effect of social security in different levels of agricultural investment. Therefore, we use the Quantile Regression method to explore the difference in the productive investment of farmers caused by the difference in the scale of farmland transfer, that is, analyze the difference in the rate of return on the productive investment of farmers caused by the scale of farmland transfer under the constraints of the comprehensive social security.

**Figure 1:**
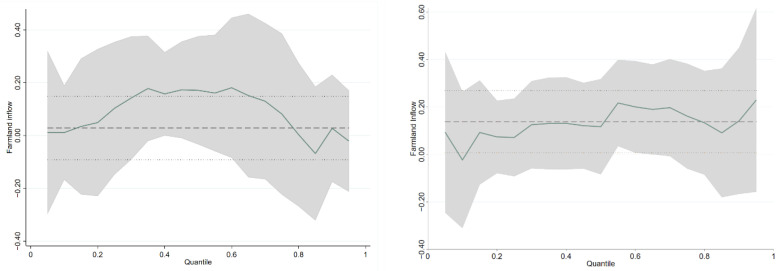
The fitting curve of farmland transfer on current investment (**left**) and fixed investment (**right**).

To avoid the issue of the zero-inflated model, we deleted the samples without current or fixed investment in Quantile Regression. We take farmer’s productive investment (current and fixed investment) as the dependent variable, take the crossover item of farmland transfer and social security level and control variables as the explanatory variable, and conduct quantile regression 99 times at the 0.01~0.99 quantile of the dependent variable. Unconditional quantile regression is used to obtain the return rate of farmland transfer to farmers’ productive investment under the constraints of social security level. We obtained the fitting curve by taking 99 quantiles as the abscissa and the regression coefficient value (partial effect or rate of return) as the ordinate (Figure 1).

Figure 1 reports the change in the farmland transfer coefficient at different quantiles in the quantile regression model. Under the constraints of social security, the farmland transfer is significantly related to the current investment of farmers. The curve of coefficients is an inverted U-shaped, with a small number of adverse effects at both ends and a positive effect in the middle. Under the constraints of social security, at quintiles where current investment is meager, such as cold and arid areas that are particularly unsuitable for agricultural production, farmers will not prioritize agricultural production after farmland inflow. They may develop a higher-profit secondary industry, decreasing current agricultural investment.

In this way, farmland transfer is negatively correlated with current investment. When the households’ current investment is low, farmland transfer will significantly positively affect farmer households’ current investment with improved social security. When the current investment level is relatively high, the farmland transfer will slightly negatively impact farmers’ current transfer with social security increases. One possible reason is that the areas with the low rural household investment are generally the central and western regions in China, where agricultural modernization is inadequate. In these areas, agricultural management conditions are poor, and the fragmentation of farmland is severe. Through farmland transfer, large-scale operations can be formed, and agricultural income can significantly increase. The expansion of rural households’ current investment provides economic conditions; the areas with higher investment are primarily concentrated in the eastern agricultural economically developed regions. After farmland inflow, farmers do not engage in relatively low-yield agricultural industries [18]. Instead, the inflowed farmland is used to develop rural tertiary sectors with relatively high returns on land, such as rural tourism and rural folk customs, which slightly negatively impacts the current transfer of rural households due to the farmland transfer.

Figure 1 also shows that under the constraints of social security, different farmland transfer scales positively affect farmers’ fixed asset investment, offering a curve of rising volatility. The result shows that under the constraints of social security, the transfer of arable land among different quantiles will lead to the expansion of farmer’s fixed investment, and the larger the scale of the inflow, the higher the amount of farmers’ fixed investment. The possible reason is that modern agricultural development in farmers with fixed agricultural investments is relatively high. The allocation of rural factors is somewhat sufficient and reasonable, and increased farm productivity and return rates can be achieved. Generally, they have solid agricultural production willingness, and families result from satisfying income from farm production and hope to increase wealth through farmland inflow. As the expansion of fixed investment is one of the necessary conditions for large-scale agricultural production, these farmers will expand fixed investment after farmland inflow. Therefore, the regression coefficient is generally positive and fluctuates slightly.

## 5. Discussion

The research conclusions above have suggestive research and policy implications. First, farmland transfer has a negligible effect on farmers’ productive investment, indicating that farmland transfer does not always result in an expansion of productive investment. This is consistent with the findings of research conducted in central China, which discovered small-scale farmland transfer does not enhance households’ productive investment [6]. Policymakers need to re-examine the expected effects of the farmland transfer policy and focus on the farmers’ worries about the future.

Second, improving social security can promote farmer households’ fixed asset investment through farmland transfer, but the impact on farmer households’ current investment is not significant and has not been fully discussed yet. The result of assessing Chinese farmers’ current productive investment behavior shows that farmland transfer requires full respect for the farmers’ will. On the one hand, improving the social security level of farmers can solve the problem of low willingness to transfer farmland and increase the expansion of farmers’ fixed investments. On the other hand, under the restriction of the social security level, the farmland transfer does not encourage farmers to increase their willingness to the current investment. Farmland transfer in China must be pre-set at a reasonable scale. It is impossible to transfer all farmland to develop modern agriculture, so disadvantaged farmers will lose the last layer of survival guarantee.

Third, most scholars believe that the improvement in the social security system may encourage farmers to increase their willingness to farmland transfer and investment [14,19]. However, this paper found that the role of social security in regulating the farmland transfer and the current investment of farmers is different in different stages of investment in agricultural production. In areas where farmers’ current investment is relatively low, it is essential to create a good policy and institutional environment. In areas with a high productive investment by farmers, it is necessary to comprehensively improve the social security system and improve the multi-dimensional survival ability of farmers.

Fourth, under the constraints of social security, the farmland transfer significantly impacts smallholder farmers’ fixed agricultural investment. According to the research, enhancing social security will hasten farmland transfer and encourage high-quality economic growth in the countryside [20]. Therefore, the construction of the agricultural socialized service market should be strengthened, and the critical role of socialized agricultural service organizations, new business entities, and significant professional households in the mechanized service of small farmers should be optimized.

This paper examines the relationship between farmland transfer, social security, and farmers’ productive investments using small farmers as the research subject and micro-data as the basis. However, this paper focuses on the current situation of China. The systems for farmland transfer vary significantly between nations. Then, due to social security limitations, how does the farmland transfer affect the household’s productive investment in various countries? The focus of future studies will primarily be on this direction.

## 6. Conclusions

Promoting the compelling connection between smallholder farmers and the development of modern agriculture is a common concern of the academic community and policy makers. This article takes small farmers as the research object. It uses the data of the Chinese Family Tracking Studies CFPS2018 as the basis to conduct a theoretical and empirical analysis on how farmland transfer affects farmers’ productive investment and the moderating role of social security. The conclusions are as follows:

First, farmland transfer has no significant impact on the productive investment of farmers (including current investment and fixed investment) without considering the comprehensive social society situation of the farmers. Second, with the expansion of comprehensive social security for rural households, farmland transfer still significantly affects the fixed investment of rural households but not their current investment. This result may be related to the subsistence guarantee function of farmland and the higher barriers to entry for fixed investment. Third, the moderating impacts of social security levels vary at different stages of households’ production investment. Under the social security level constraints, there is an inverted U-shaped relationship between farmland transfer and rural households’ current investment. Specifically, when current investment is at a low and high level, as comprehensive social security increases, farmland transfer will harm households’ current investment. When the rural household’s current investment scale exceeds the critical point, farmland transfer will positively impact the current investment by improving the level of social security. In addition, for the fixed investment of rural households, regardless of the status of their investment, the partial effect of farmland transfer on fixed investment is significantly positive and exhibits a tendency of rising volatility.

## Figures and Tables

**Table 1:** The description of variables.

Variable		Mean	Std. Dev.	Variable Description
Dependent variables	*ave_fxdinvest*	2.3039	3.0354	Fixed investment per land(log)
	*ave_curtinvest*	4.8711	3.0015	Current investment per land (log)
Explanatory variable	*inflow_farmland*	0.0050	1.0356	The scale of farmland inflow
Family endowment Farmland endowment	*outflow_farmland*	0.0023	1.0334	The scale of farmland outflow
	*compr_socialsec*	0.0047	1.0089	The score of comprehensive social security
	*max_age*	−0.0069	0.9996	The max-age of the family members
	*relative_income*	0.0035	0.9974	Household income percentile
	*edu_expd*	0.0072	1.0129	Family education expenditure in the past year
	*stru_expd*	0.0783	0.0020	Agricultural expenditure to the total family expenditure
	*family_size*	−0.0059	0.9976	The number of family members
	*ave_farmland*	0.0029	1.0399	Average scale of farmland for a family member
	*land_asset*	0.0061	0.9738	The average land asset for a family
	*stru_land*	−0.0090	0.9832	Paddy and dry farmland to total agricultural land
	*optd_farmland*	−0.0001	0.8701	The scale of operated farmland of the family
Village endowment	*disaster_zone*	−0.0025	0.9993	Whether the village is in a natural disaster zone, 1 is yes, 0 is no
	*dist_county*	0.0144	1.0158	The distance to the nearest county
	*inner_road*	−0.0002	1.0021	The proportion of dirt roads
	*mig_work*	0.0058	1.0036	The proportion of migrant workers
	*stru_industry*	−0.0076	1.0401	The proportion of the output value of primary industry
	*max_clan*	0.0045	1.0003	The population rate with the most prominent surname
	*stru_income*	0.0548	0.0239	The proportion of agricultural income in total income

**Table 2:** The regression result of farmland transfer on farmer’s productive investment.

Variables	*ave_curtinvest*	*ave_fxdinvest*	Variables	*ave_curtinvest*	*ave_fxdinvest*
*inflow_farmland*	0.0328	0.0347	*disaster_zone*	0.1008	0.2052 ***
*outflow_farmland*	−0.0347	0.0117	*dist_county*	0.0407	−0.1244 **
*max_age*	−0.1871 ***	−0.2477 ***	*inner_road*	0.2087 ***	0.0743
*relative_income*	0.1091 *	−0.0394	*mig_work*	−0.3528 ***	−0.2196 ***
*edu_expd*	0.0011	0.1036 *	*stru_industry*	0.0185	−0.1994 ***
*stru_expd*	−24.5431	−7.9000	*max_clan*	−0.0557	−0.0081
*family_size*	0.3168 ***	0.5063 ***	*stru_income*	−0.6098	0.7621
*ave_farmland*	−0.2600	−0.2920	_cons	4.2558 **	5.4884 ***
*land_asset*	0.2102 ***	0.1853 ***	Adj.R^2^	0.0592	0.0430
*stru_land*	0.1009	0.0282	Observations	4703	4703
*optd_farmland*	−0.0245	−0.1717 ***	—	—	—

Note: *, **, *** represents the 90%, 95%, and 99% confidence level and below.

**Table 3:** The zero-inflated Poisson regression result.

Variables	*ave_curtinvest*	*ave_fxdinvest*	Variables	*ave_curtinvest*	*ave_fxdinvest*
*inflow_farmland*	0.0056	0.0366 *	*disaster_zone*	0.0126 ***	−0.0034
*outflow_farmland*	0.0059	0.0029	*dist_county*	−0.0124 ***	0.002
*max_age*	−0.0098 **	−0.0083	*inner_road*	−0.0067 *	−0.0191 **
*relative_income*	0.0256 ***	0.0338 ***	*mig_work*	−0.0239 ***	−0.02 ***
*edu_expd*	−0.003	−0.0146 **	*stru_industry*	−0.0064 **	−0.0046
*stru_expd*	−0.4725	1.1749	*max_clan*	−0.013 ***	−0.0027
*family_size*	0.0223 ***	0.0237 ***	*stru_income*	0.0254	−0.2381 ***
*ave_farmland*	−0.0603 ***	−0.0145	*Inflow* × *socialsec*	0.1247 ***	1.7026 ***
*land_asset*	0.0251 ***	−0.0043	_cons	1.8886 ***	1.7026 ***
*stru_land*	0.005	0.026 ***	*compr_socialsec*	0.0951 *	0.0707 **
*optd_farmland*	−0.133 ***	−0.1581 ***	Vuong (z)	125.47	113.12

Note: *, **, *** represents the 90%, 95%, and 99% confidence level and below.

## Data Availability

Not applicable.
